# Supplementary material for: Testing, tracing and isolation in compartmental models
Source: PLoS Comput Biol. 2021 Mar 4;17(3):e1008633. doi: 10.1371/journal.pcbi.1008633 (PMC7932151; doi:10.1371/journal.pcbi.1008633)
Supplement: Attachment — Submitted filename: Reproducible_report_PCOMPBIOL_D_20_00860_signed.pdf [file pcbi.1008633.s001.pdf]

Director: Professor Herbert M. Sauro
University of Washington, Seattle, WA
https://reproduciblebiomodels.org

Reproducibility report for: Testing, tracing and isolation in compartmental models
Submitted to: PLoS Computational Biology
Manuscript number/identifier: PCOMPBIOL-D-20-00860

Curation outcome summary: Following the documentation available in the Github repository provided by the authors we were unable to reproduce the simulation results presented in this submission. The model source code could, however, be utilized by the curator in developing new scripts to produce some of the data presented in this submission.

Box 1: Criteria for repeatability and reproducibility

- [x] **Model source code provided:**
  - [x] Source code: a standard procedural language is used (e.g. MATLAB, Python, C)
    - [x] There are details/documentation on how the source code was compiled
    - [x] There are details on how to run the code in the provided documentation
    - [ ] The initial conditions are provided for each of the simulations
    - [x] Details for creating reported graphical results from the simulation results
  - [ ] Source code: a declarative language is used (e.g. SBML, CellML, NeuroML)
    - [ ] The algorithms used are defined or cited in previous articles
    - [ ] The algorithm parameters are defined
    - [ ] Post-processing of the results are described in sufficient detail
- [ ] **Model source code not provided:**
  - [ ] The model is executable without source (e.g. desktop application, compiled code, online service)
    - [ ] There are sufficient details to repeat the required simulation experiments
- [x] **The model is described mathematically in the article(s):**
  - [x] Equations representing the biological system
  - [x] There are tables or lists of parameter values
  - [ ] There are tables or lists of initial conditions
  - [ ] Machine-readable tables of parameter values
  - [ ] Machine-readable tables of initial conditions
- [x] **The simulation experiments using the model are described mathematically in the article:**
  - [ ] Integration algorithms used are defined
  - [x] Stochastic algorithms used are defined
  - [ ] Random number generator algorithms used are defined
  - [ ] Parameter fitting algorithms are defined
  - [ ] The paper indicates how the algorithms yield the desired output

Box 2: Criteria for accessibility

- ■ Model/ source code is available at a public repository or researcher's web site
  - ☐ Prohibitive license provided
  - ☐ Permissive license provided
  - ■ Open-source license provided
- ☐ All initial conditions and parameters are provided
- ☐ All simulation experiments are fully defined (events listed, collection times and measurements specified, algorithms provided, simulator specified, etc.)

Box 3: Rules for Credible practice of Modeling and Simulation[a]

[a]Model credibility is assessed using the Interagency Modeling and Ananlysis Group conformance rubric: https://www.imagwiki.nibib.nih.gov/content/10-simple-rules-conformance-rubric

- ■ Define context clearly: Extensive
- ■ Use appropriate data: Adequate
- ■ Evaluate within context: Extensive
- ■ List limitations explicitly: Adequate
- ■ Use version control: Adequate
- ■ Document adequately: Adequate
- ☐ Conform to standards: Insufficient

Box 4: Evaluation

- ■ Model and its simulations could be repeated using provided declarative or procedural code
- ☐ Model and its simulations could be reproduced

Summary comments: Implementation of the model reported in this submission was provided as Python code available for download from a Github repository. This was used in our attempt to reproduce the simulation results reported in this manuscript. Running the sample "ptti" commands we were able to produce the example plots reported in the source code documentation. We were unable to successfully run the suggested benchmark commands to reproduce the results from this manuscript as reported in the Github documentation. We were, however, able to use the programmatic interface of the model codes as well as the "ptti" module to reproduce some of the results presented in Figures 2, 4, 6 and 7 from Python scripts written by the curator. Specifically, we were able to produce curves associated with both the ODE-based and ABM simulations using information found in the .yaml files of the benchmarks folder. This leads us to believe that with some clarity in the use of the provided code and its distribution, this submission would be reproducible.

Anand K. Rampadarath[1], PhD
Curator
Center for Reproducible Biomedical Modeling

David P. Nickerson, PhD
Curation Service Director
Center for Reproducible Biomedical Modeling

Auckland Bioengineering Institute,
University of Auckland

[1] Email: a.rampadarath@auckland.ac.nz
