# Supplementary material for: Testing, tracing and isolation in compartmental models
Source: PLoS Comput Biol. 2021 Mar 4;17(3):e1008633. doi: 10.1371/journal.pcbi.1008633 (PMC7932151; doi:10.1371/journal.pcbi.1008633)
Supplement: Attachment — Submitted filename: PLoSCompBiol_Response_Editor_Reviewers.docx [file pcbi.1008633.s002.docx]

Dear Editor and Editorial Board,

We thank the Editors and the Reviewer for their insightful and thorough comments on our manuscript. We have now gone through the comments and revised the manuscript accordingly. Below, in blue, are our responses to reviewers’ comments and suggestions, noting the changes we have done in response to the comments.

In addition, to improve the flow of text and accommodate the responses to the reviewers, we have also revised the manuscript. The main changes to the manuscript are listed at the beginning of the manuscript version with highlighted changes.

**Editors’ comments:**

We apologize for the delay in our decision. It was extremely challenging to find experts who had the capacity or the willingness to review your work. Unfortunately, we ended up with only one scientific review report. This report is, in our view, insightful and generally positive. Because we, the editors are also positive about your work we decided to take a decision with fewer reports than usual.

We thank the Editors for being positive about our work.

It would be great if you would address the points of criticism the reviewer raises, and that we believe are well taken. Especially the point on extending your perspective of ABM by more realistic infectivity profiles and distributions of time spent in each state would be worthwhile to discuss as a limitation. We also encourage you to include a comparison with an ABM that makes more realistic assumptions in this regard, if feasible.

In response to the reviewer’s comment (detailed below), we have included an additional ABM using a different distribution of event timings rather than the rates of an exponential distribution. We have undertaken analysis comparing this, more complex, ABM with the SEIR-TTI and reported the findings in the manuscript.

In addition, there is previous work by Fraser et al that you mention in the Introduction (ref 52) that is very similar in scope to your work. It would strengthen your work, in our opinion, if you compared your work to the approach by Fraser et al in more detail in the Discussion.

We agree with the Editors and we have now included an additional paragraph on comparisons of our model to that of Fraser et al. in our Discussion (pp 16 (in clean revised copy)). We do note that the McKendrick-Von Foerster equation that Fraser et al. use in their framework, is a slightly different concept. Under the assumption that the modelled process is a Markov process and infections from individuals that occur at a rate that only depends on the current state, the McKendrick-Von Foerster equation becomes a system of ODEs but different to the one we use. In the discussion we have highlighted this and we believe that both of the studies should independently stand next to each other and complement one another.

Lastly, please also note the suggestions in the reproducibility report to improve documentation and reporting of initial conditions.

We are very much in favour of reproducible science and are pleased to participate in this process. We were encouraged by the positive reproducibility report. To make our work fully reproducible, we now have a branch called ptti-theory-paper available at our GitHub repository <https://github.com/ptti/ptti/tree/ptti-theory-paper> that contains all the material to reproduce the results and, specifically the Figures of this manuscript.

**Reviewer #1: The Reproducibility Report is uploaded as an attachment.**

We thank the reviewers for the reproducibility report. In order to facilitate easier reproducibility of the results, we have produced a branch of our repository in GitHub called ptti-theory-paper and available at <https://github.com/ptti/ptti/tree/ptti-theory-paper>. This branch contains all the material to reproduce the results and, specifically the Figures in this manuscript. The branch has also been explicitly mentioned in the manuscript (pp 15, 2nd paragraph) and will not be subject to further changes.

Reviewer #2: In this work, Sturniolo et al. formulate a method for modelling contact tracing in a compartmental model and test this ODE approximation against an ABM with the same structure. Overall, I think this work is valuable and should be published. I particularly liked the clear discussion of when the ODE approximation breaks down.

We thank the reviewer for their positive comments.

That said, I do have one major comment relating about the impact of constant infectivity and constant transition rates in ODE models. Ideally, this would require additional comparison between the ODE model and ABM. If this involves a prohibitive amount of work, it could potentially be adequately addressed through additional discussion.

In light of this comment from the reviewer, we have extended our analysis to include an additional ABM using a different distribution of event timings rather than the rates of an exponential distribution. The distributions in this ABM are chosen to show a limit-case comparison with SEIR-TTI.

I also have some additional points relating to presentation.

1. Exponential time distributions in ODE and ABM

The advantage of ABMs is discussed in terms of allowing fine-grained modelling of individual variation in susceptibility to disease and contact patterns. The authors point out that we often lack data to parametrise such fine grain models.

However, in the context of contact tracing, ABMs provide additional flexibility for important parameters we do have data about:

i) more realistic infectiousness profiles than the constant infectiousness assumed in ODE models (for covid infectiousness profiles, see for example He et al., 2020 Nature Medicine; Ferratti & Wymant et al, 2020 Science);

ii) more realistic distribution of times spent in each state – for example, fixed time delays for testing and tracing rather than constant rates (which lead to exponential waiting time distributions).

We agree with the reviewer on these points of the advantage of ABMs over ODEs and our group works actively on both types of models; see for example Panovska-Griffiths et al. *Lancet Child Adolesc Health* 2020; 4:817-27 for a fine-granulated ABM applied to the COVID-19 epidemic.  The additional ABM that we have now included for comparison features fixed time-delays for comparison. We have also included, slightly rephrased, the comments that the reviewer made in our discussion (pp 17).

These are of course standard limitations of ODEs, but I think it may be particularly important when the aim is to model contact tracing: the effectiveness of tracing depends on where in the infectiousness profile individuals are isolated. The interplay between assumptions about the shape of the infectivity profile and waiting time distributions may therefore have a significant impact.

We agree with the reviewer the shape of the infectivity profile and waiting time distributions may have a significant impact and our second ABM allows exploration of this. We also note that one of the key public questions associated with the pandemic is compliance to isolation vs contact tracing. This is why we have within the paper explored testing and isolation as one strategy (pp 4) and test-trace-isolate (pp 5) as another. In addition in a follow-on work we will look at the trade-offs between compliance to isolation and the length of the isolation period - but this work is beyond the scope of this paper.

This manuscript would be considerable stronger if the authors included a comparison with an ABM with different assumptions of infectivity profile and time distributions (even for just one of the processes – e.g. the time it takes to trace contacts), to give a reader a sense of the magnitude and direction of the error this may introduce. However, if implementing such a comparison is a prohibitive amount of work, discussing this point might be enough, particularly if the authors are able to provide an intuition for how these approximations might affect the output of the ODE.

We thank the reviewers for identifying this important point. To address this, we have developed and added a modified version of our ABM model to the manuscript (pp 9) and also added the code for the model in the relevant GitHub repository. The new method represents an extreme limit of a typical bell-shaped distribution for the times, with a single fixed time for each transition. Simulations done with this model have been performed, and they show a good match for the high testing regime, and they predict higher infection levels than the ODEs for low testing instead. The relevant theory has been added to the text in the Methods section of the manuscript (pp 9). In addition two additional figures were also added (Figures 8 and 9) that illustrate the performance of this model and corresponding results in the Results section (pp 5-6).

We have also added a sentence to the discussion regarding choices of distribution (pp 17) for both the progression of the disease (where a peaked distribution is realistic) and the contact events mediated by a contact network (which can produce very different distributions). Neither of these distributions are the exponential distributions implied by well-mixed models.

2. Framing of testing and interpretation of theta

In the results section, testing and isolation (without tracing) is presented as random testing of the “entire population” (but also, confusingly, “only infectious individuals are tested and isolated”). Theta is therefore interpreted as the average testing rate in the population. In the methods, the same process is described as infectious individuals experiencing symptoms and seeking diagnosis. Theta is therefore interpreted as the rate of experiencing symptoms and receiving a diagnosis.

Firstly, this inconsistency makes the paper confusing to read.

Secondly, it is not clear to me that that these two framings of theta are equivalent: if the entire population gets tested every 14 days on average, the expected time delay between getting infected and tested would be 7 days.

We thank the reviewer for pointing out this inconsistency and they are correct that the two interpretations are not necessarily equivalent. Indeed, the interpretation from the methods is the correct one. We have now changed the text in the results section to reflect this (in Figure 4) and it is now consistent.

I think the authors are aware of this, based on the last paragraph on page 6 (or is theta = 1/14 a typo? The value of theta is 1/7 in subsequent figure legends), so it is not clear to me whether I should interpret the result that “testing the entire population every 20 days” as an expected delay from infection to diagnosis of 20 or 10 days.

This is an error in the paper, unrelated to the above confusion. The correct value for those figures is 1/14. This is chosen, as explained in the text, to be slower than the recovery time in order to illustrate the amplification that the contact tracing produces. The interpretation is, in fact, that they experience a delay of 20 days. This effectively means that many people will not be tested and that testing on its own would be ineffective, but that even such a low testing rate can help mitigate the epidemic with good tracing. We have corrected this to state 1/14 and also added a clarification in the text of Figure 4.

3. Minor points:

- Calling the D compartment “diagnosed” is confusing when it also applies to susceptible individuals. In particular, this makes figure 1 difficult to interpret.

We agree with this point. Indeed a better name for this compartment might be “misdiagnosed”. However changing this throughout would be very difficult as it would mean changing the figure, many of the equations, and the software, and would achieve but a small benefit. While we agree with the reviewer’s point, doing so while ensuring consistency would be too time costly and we don’t feel this labelling distracts from the point and the context of the paper. We have therefore decided to leave this as in the original submission, but to clarify, we have pointed out the possibility of misdiagnosis through tracing in the caption of Fig 1.

- Some variables are not defined when introduced (eta in equation 1, C_I in Figure 1).

We apologise for this. Eta is now defined when used and C_I is now explained; we don’t believe there are any other outstanding unlabelled variables.

- R stands for both recovered and reproductive number, which makes figures 2 and 4 a little confusing (from the axes labels, one would assume R(t) is recovered).

We agree with this reviewer’s point and to make this difference clear we have now labelled the effective reproduction number Re(t) in Figures 2 and 4 and throughout the manuscript text.

**Have all data underlying the figures and results presented in the manuscript been provided?**

Large-scale datasets should be made available via a public repository as described in the *PLOS Computational Biology* [data availability policy](https://eur01.safelinks.protection.outlook.com/?url=http%3A%2F%2Fjournals.plos.org%2Fploscompbiol%2Fs%2Fdata-availability&data=02%7C01%7C%7C67f8317e5c74473f3df608d8662d6be5%7C1faf88fea9984c5b93c9210a11d9a5c2%7C0%7C0%7C637371692876785391&sdata=dN0YNxoTH%2FdxOnJGXM3uid0JZhlfxSY%2Ffyw5tJQNIFg%3D&reserved=0), and numerical data that underlies graphs or summary statistics should be provided in spreadsheet form as supporting information.

Reviewer #1: None

Reviewer #2: Yes
